# Solving self-absorption in fluorescence

**DOI:** 10.1107/S2052252519005128

**Published:** 2019-05-10

**Authors:** Ryan M. Trevorah, Christopher T. Chantler, Martin J. Schalken

**Affiliations:** a School of Physics, University of Melbourne, Australia

**Keywords:** fluorescence, X-ray absorption fine structure (XAFS), X-ray absorption spectroscopy (XAS), self-absorption, software and modelling

## Abstract

One of the most common types of experiment in X-ray absorption spectroscopy (XAS) measures the secondary inelastically scattered fluorescence photon. This widespread approach has a dominant systematic of self-absorption of the fluorescence photon. The large impact of self-absorption compromises accuracy, analysis and insight. Presented here is a detailed self-consistent method to correct for self-absorption and attenuation in fluorescence X-ray measurements. This method and the resulting software package can be applied to any fluorescence data, for XAS or any other experimental approach detecting fluorescence or inelastically scattered radiation, leading to a general solution applicable to a wide range of experimental investigations. The high intrinsic accuracy of the processed data allows these features to be well modelled and yields deeper potential insight.

## Abstract 2

Equations and methodology for correcting the dominant systematic in fluorescence measurements are provided. They are particularly appropriate for X-ray absorption spectroscopy in fluorescence and for multi-pixel detection.

## 1. Introduction   

X-ray absorption fine structure (XAFS) is the oscillatory behaviour in photoelectric X-ray absorption spectra above an ionization edge. The oscillations are caused by backscattering and self-interference of the wavefunction of an emitted photoelectron within the material near the emitting atom. From these oscillations, we can extract highly accurate information on the local atomic structure surrounding the X-ray absorbing atom. XAFS is one of the most popular techniques used in synchrotron measurements and applications have been found in many diverse fields: fundamental physics (Bertoni, 2015 ▸), pure and applied chemistry (Lamberti & Bokhoven, 2016 ▸; Islam *et al.*, 2016 ▸), biological and medical science (Fornasini, 2015 ▸), earth sciences and engineering (Boscherini, 2015 ▸; Ramaker, 2016 ▸), and art and cultural heritage (Farges & Cotte, 2016 ▸). However, the potential of this technique is often limited by poorly quantified experimental uncertainties or untreated systematic effects (Creagh & Hubbell, 1987 ▸, 1990 ▸; Krappe & Rossner, 1999 ▸). Fluorescence measurement, developed by Jaklevic *et al.* (1977 ▸), is a particularly useful technique for dilute systems (Jaklevic *et al.*, 1993 ▸; Lee *et al.*, 1981 ▸) and is very commonly used for modern experiments. For XAFS measurements conducted using fluorescence detection, there is particular difficulty in obtaining accurate statistical uncertainties compared with experiments conducted in ‘transmission mode’, and most publications are reported with no uncertainties.

One of the earliest attempts to explain the distortion of fluorescence X-ray absorption spectroscopy (XAS) was made by Goulon *et al.* (1982 ▸). They recognized the key limitations of fluorescence measurement from the loss of statistics in the signal-to-noise ratio, some attenuation of both the edge jump and the XAFS oscillations, the fluorescence yield, the solid angle and the core integral.

The first studies to attempt to correct for self-absorption in fluorescence detection of XAFS were reported in a series of papers by Troger *et al.* (1992 ▸). They commented that fluorescence-yield XAFS has particular advantages in many cases due to its clean signal over the background and a much higher information depth or effective depth compared with electron-yield measurements. They noted that in two ideal limits, of thin films or dilute samples, the intensity is directly proportional to the central atom absorption coefficient of interest and therefore, in principle, the extraction of the XAFS may be straightforward. In concentrated or ‘thick’ samples, they noted that the absorptive effects distort the signal dramatically, and errors in the determination of physical parameters can exceed 50%. They considered the solid angle, core integral and fluorescence yield. Troger *et al.* (1992 ▸) illustrated a partial correction for small amplitudes of oscillation and absorptive corrections but only in χ, and with significant distortions remaining.

Previous investigations of these distortions in fluorescence spectra of XAFS have required knowledge of the sample stoichiometry (Troger *et al.*, 1992 ▸), making a series of measurements at multiple angles (Eisebitt *et al.*, 1993 ▸) or knowledge of the known energy dependence of the absorption coefficients (Pfalzer *et al.*, 1999 ▸). These studies could partially correct for absorptive effects. Prior to 2005, studies assumed that ‘the effect of the XAFS on the correction term was very small’ and that ‘the samples are in the thick limit’, as stated by Booth & Bridges (2005 ▸). Those authors correctly state that the derivation or inversion of the constitutive equations, required to obtain a coefficient which can be analysed by theory or software packages, can be challenging because there is a singularity, so that in some general limits the inversion does not exist. They investigated a sample (4.9 µm Cu metal foil) closer to a thicker limit where the inversion effect was strong (μ*t* > 1), and performed an inversion using an expansion from the ‘thick dilute sample limit’ combined with a thin-limit first-order expansion of the key term in the exponential.

Other problems with past work include: (i) the critical need to consider the beam path for the incident and fluorescent photons to invert or obtain a true absorption coefficient and thereby make a clear link with theory; (ii) the use of absorption coefficients in critical terms where attenuation coefficients are needed (Chantler *et al.*, 2012 ▸); and (iii) the need to relate the analysis to the observed or measured (μ/ρ)(ρ*t*) as the primary output of the theory and hence of the fit, rather than a spline-distorted χ. Nonetheless, this work builds upon and extends the previous efforts to a more general and effective solution.

The current popular packages used worldwide for some 1000 publications of XAS per annum, such as *IFEFFIT* (Newville, 2001 ▸) and *Athena* (Ravel & Newville, 2005 ▸), do not include any such inversion for (μ/ρ) and instead follow some of the above work in a particular adaptation of a χ inversion, together with the limitations mentioned above. An older program, *FLUO*, deals with a limited application of self-absorption correction to XANES, whereas some of the latest packages developed such as *Larch* (Newville, 2013 ▸) do not include any inversion at all. It is high time to improve upon this, and to implement a robust and user-accessible approach. We hope that these and other packages will incorporate this analysis in a routine manner.

## 2. A key problem in general X-ray fluorescence analysis   

**Figure 1:**
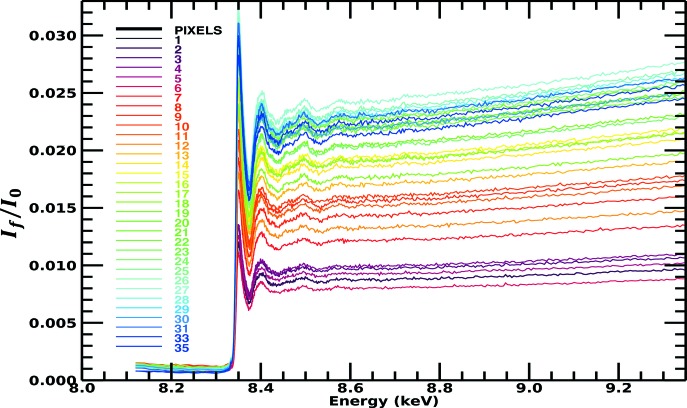
Raw fluorescence spectra for the nickel *i*-pr complex, demonstrating the large dispersion between spectra collected by individual pixels. This is well known and common in XAS fluorescence-mode data collection.

A dramatic discrepancy between raw spectra from transmission and raw spectra from fluorescence measurements is well known, as exemplified by Figs. 1 ▸ and 2 ▸ for the Ni complexes analysed in this study. There is a large and divergent dispersion between the individual fluorescence pixel spectra, and the spectral shape is distorted, impairing high-accuracy analysis. The slope for the absorption or attenuation coefficient above the edge decreases with energy as predicted by theory, while the slope in experimental fluorescence spectra usually goes up with energy. Individual pixels in a multi-pixel fluorescence detector display different slopes. This is well understood in a qualitative sense, particularly because of the self-absorption systematic, attenuation and uncalibrated detector efficiencies in fluorescence. While the detector efficiency can be corrected for, XAFS needs a self-consistent method for removing the effect of self-absorption from the spectra. The distortions due to attenuating and self-absorptive effects are particularly serious, as stated by Troger *et al.* (1992 ▸), because they will lead to different parameterized fits, *i.e.* different structural and model parameters, compared with a corrected spectrum following the theoretical predictions for absorption (transmission). With the data set illustrating this discussion (Fig. 1 ▸), there is a particularly strong variation in the pixel-dependent amplitude, in part from the significant variation in the pixel-dependent horizontal and vertical solid angles across the detector relative to the sample.

Previous attempts to remove this systematic have not significantly reduced the variance between pixel spectra or different angles and have not produced a physical trend of (μ/ρ) with energy. Any measure of variance (a combination of statistical and systematic point-wise uncertainty) was dominated by the large pixel- and angle-dependent variations of all sources, rather than defining the consistency and reliability of the data, statistical or otherwise. The high-accuracy analysis of many important data sets for chemistry, bonding, biological dynamics and disease is therefore limited, with many data sets partially analysed, unpublished or abandoned.

In this work, we predict to high accuracy the magnitude of the dispersion and the energy functional due to self-absorption. We present the software package *SeAFFluX* (self-absorption fitting of fluorescent X-rays) to correct for this systematic in a self-consistent and robust manner. We invert the effects of self-absorption and attenuation to extract (μ/ρ) directly, using alternate approximation methods, allowing a direct comparison with theory and with transmission experiments without additional processing. As a result, the dispersion is dramatically reduced, and the shape and pattern for each pixel or angle are consistent. Further, the corrected spectral shape follows the required trend for absorption and the absorption coefficient, and can thereby be related directly to theory. Hence, the pixel variance provides a reliable estimate of statistical and some of the systematic uncertainty.

In the supporting information, we provide: (i) software able to predict and correct for the self-absorption systematic using real experimental fluorescence data; (ii) the installation and operational manual for said software; and (iii) tabulations of 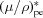 versus *E* for two 15 m*M* Ni complexes from fluorescence measurements in two formats, namely a prototype error-propagating input suitable for *eFEFFIT* (Schalken & Chantler, 2018 ▸) and *IFEFFIT* input, and a prototype IUCr CIF data format.

## 3. Applications and limitations of model, software and experiment   

The complexes considered in this work have local metal environments with approximate tetrahedral and square-planar coordination geometries (Fox *et al.*, 1964 ▸; Britton & Pignolet, 1989 ▸). A previous publication has confirmed these structures using transmission-mode XAFS (Chantler *et al.*, 2015 ▸). This system therefore provides an excellent test of fluorescent multi-pixel data and processing (Best *et al.*, 2016 ▸; Islam *et al.*, 2017 ▸). We chose this system explicitly because it is a crucial application typical of many XAFS samples and systems, because the equations and solutions generalize to many more, and because high-quality transmission spectra (using the X-ray extended range technique, XERT) and high-quality fluorescence data are obtained for the same sample in simultaneous data collection. It thereby proves the success and efficacy of the approach and functional.

For highly attenuating samples, transmission mode has poor statistics or becomes infeasible. In such cases fluorescence-mode detection is excellent but it cannot be directly compared with a corresponding absorption coefficient or a reference transmission experiment. For extremely low attenuation, the transmission statistics become very challenging and the clean signal to background of fluorescence is generally a preferred option, yet once again direct comparison with an absorption coefficient and the prediction of theory is lost. We choose an experimental illustration herein where high accuracy is possible but not easy by transmission, and where it is possible also by fluorescence measurement, so that a direct comparison can be made.

The principles of our model apply to all geometries of fluorescence. Most fluorescence data are collected with the sample at 45° to the incident beam, where the measurement is most sensitive to bulk sample properties but also where the angular variation of self-absorption is very significant. The detector is typically perpendicular to the incident beam. Some groups use somewhat specialized techniques of normal incidence–grazing fluorescence or grazing incidence–normal fluorescence geometries. Both these geometries lead to (much) greater attenuation and hence lower signal, and to the techniques being primarily surface techniques. Penetration depths in these grazing cases may reach as high as micrometres or may be only a few nanometres, compared with standard geometry depths reaching 20 µm or more. The grazing incidence–normal fluorescence and related geometries have very large self-absorption effects and distortions and the discussion here remains relevant. The normal incidence–grazing fluorescence geometry reduces the self-absorption *per se* but can still have significant contributions (Pease *et al.*, 1989 ▸). Our model and software apply to all possible angles except directly to the reflection XAFS geometry, where the reflectivity coefficients require significant additional discussion. A key advantage of the model and software is that they apply to ranges of experimental angles of incidence and especially fluorescence relative to different pixels of a multi-pixel detector, and the experimental evidence of the multiple elements is used directly to assess the correction of absorptive effects relative to the intended absorption coefficient from transmission experiments. In a similar vein, electron-yield measurements are based upon the inelastic cross section and a possible extraction potential, but of course would require different code for the electron ‘self-absorption’ cross sections and treatment.

The example we present here involves the standard positioning of the fluorescence detector. A major question relates to the investigation of *k* range, and in particular whether the investigation relates to pre-edge analysis, edge profile, XANES structure, conventional XAFS *k* ranges *etc*. Dispersion between pixels and angles is more significant at medium and higher *k*, and less significant towards low *k*. Equally, the largest effects of self-absorption and attenuation are around the white line and for low-*k* regions. So the most critical areas which this analysis and code address and fix relate to low-*k*, medium-*k* and high-*k* and they should address all of these regions. In the pre-edge, there are major theoretical issues which probably prevent any direct interpretation by theory or following a transmission and absorption coefficient at this time, and further work will be needed to explore this (Chantler, 2019 ▸). In the edge profile there are certainly additional systematics which are not addressed by this processing; however, the approach and software should apply to all XANES and XAFS analysis. In particular, we provide one general inversion expansion appropriate for the edge and near-XANES region and one that is more accurate for the longer XANES and XAFS region. Both are efficacious.

Typical samples can approach three limits: thin samples, thick dilute samples or thick concentrated samples. The ‘thin limit’ has relatively minor corrections due to self-absorption and attenuation effects, so while the current approach and software are valid there may be only a small impact upon the final structure determination and analysis. If the multiple pixels display identical spectra, curves, offsets and slopes, then the corrections are likely to be minor or the detector is at a small solid angle to the sample. If the slopes look identically like an absorption coefficient spectrum from a transmission experiment, then the corrections are minor, but the approach and software remain valid. However, even in the thin limit the corrections and dispersion represented below can be large and important for fluorescence analysis. Typical examples might be thin unsupported samples below 1–5 µm or ultra-dilute thin solution cells, *e.g.* less than a millimetre in depth.

Samples close to the ideal ‘thick limit’ would be very thick copper foils, thick concentrated solutions with a high molarity of metal atoms, or thin samples supported by a glass slide or strongly absorbing support backing, *e.g.* for a microprobe. This last case is fully covered by the approach and software presented, in that the integrals do not need to include the backing or support slide because they should not contribute to the fluorescence signal. Of course, some support backing or matrix materials can fluoresce at the absorption edge of the active species, and if so must either be avoided (preferred) or corrected for. While measurements in the thick ideal limit are difficult or impossible to compare directly with a transmission experiment, the corrections prescribed here are robust.

The other two ‘thick’ limits can be realized by: (i) moderately thick copper foils; or (ii) moderately thick solution cells with a certain molarity (*e.g.* 0.01–25 m*M*) of active metal atoms. The example of Booth & Bridges (2005 ▸) corresponds to the former interesting limit and our example herein corresponds to the latter limit. Any intermediate samples would also be strongly distorted by self-absorption and attenuation, and all samples may be strongly affected by the dispersion effects noted and regularly observed. Such limits can be categorized by the total attenuation (μ/ρ)(ρ*t*) of the sample with a matrix, for example, and similarly by the component relating only to the photoabsorption from *e.g.* the *K* edge of interest from the metal atoms producing a fluorescence photon, 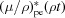. In the first ‘thick’ limit, the two measures are almost identical, and in the second limit, the first measure can be very large while the second may be much smaller. In both limits, large self-absorption and attenuation effects are observed, large dispersion is observed between angles and pixels, and the slopes should be dramatically distorted. The approach and software presented here apply to both these limits. The example given by Booth & Bridges (2005 ▸) relates to (μ/ρ)(ρ*t*) ≃ 1 immediately above the edge, whereas our example herein has (μ/ρ)(ρ*t*) ≫ 2.7 above the edge. Conversely, the example of Booth & Bridges (2005 ▸) relates to 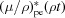 ≃ 1 immediately above the edge, whereas our example here has 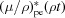 ≃ 0.1 above the edge. Another way of characterizing the magnitude of these effects is to define χ at the first peak or so above the edge. By this measure, the example of Booth & Bridges (2005 ▸) relates to χ ≃ 0.2, whereas our example herein uses χ ≃ 0.5.

In general, care should be taken to ensure that the approximations, corrections and inversion are suitable for the sample and geometry investigated. In the examples presented, we use a Taylor series expansion in the detailed software *ansatz* with a limit of convergence of order χ ≃ 1 and an expansion of the exponential suitable across most samples. One must be somewhat careful of the convergence and impact of the treatment of the exponential for very thick samples, as discussed below. Our self-absorption approach opens up new opportunities for insight from any fluorescence data on any beamline.

## 4. Experimental   

Fluorescence and transmission XAS measurements were taken simultaneously for two closely related organometallics, bis(*N*-*n*-propylsalicyladiminato)nickel(II), herein denoted *n*-pr, and bis(*N*-*i*-propylsalicyladiminato)nickel(II), denoted *i*-pr, at the Australian National Beamline Facility, Tsukuba, Japan. Solutions of each complex (15 m*M*) were prepared using 60% butyronitrile (BCN) + 40% acetonitrile (ACN) as the solvent to avoid microcrystallization at cryostat temperatures, *ca* 80 K. The exact concentrations of the solutes were 15.33 (6) m*M* and 15.26 (3) m*M*, respectively, corresponding to approximately 0.1% *w*/*w* or 1000 p.p.m. (parts per million) of nickel in the sample.

The beam was incident upon the sample at 45°. Sample (cell) thicknesses were 1.9577 (17) mm (*i*-pr) and 1.981 (2) mm (*n*-pr) [Table 3, column 4 in the report by Chantler *et al.* (2015 ▸)]. The frozen solutions were contained in cells fashioned from a 25 × 2 mm Teflon pellet, designed to allow a 1.5 × 2 mm X-ray beam to pass through. Kapton adhesive tape and a light film of silicone grease were used to minimize the risk of the solution leaking between cells. The distance between the sample and the fluorescence detector elements is 107 (2) mm. A detailed schematic of the transmission experiment with accurate distances can be found in Fig. 5 of the work by Chantler *et al.* (2015 ▸). Table 1 ▸ provides the overall experimental variables for the extremely important optical path elements upstream and downstream of the sample.

A 36-element Ge planar detector (EURISYS EPIX 36-64-7-ER) was used to collect the fluorescence. The detector contains 6 × 6 channels forming a square area, with each pixel capturing an area of 8 × 8 mm. The output file runs from channels 0 to 35, with 0 being the top downstream end and incrementing horizontally. The approximate gap between the active area of each channel is 0.4 mm, so the separation of pixel centres at the detector surface is 8.4 mm. The central position of the detector is aligned to be ∼45° to the solution cell or ∼90° to the incident beam. Three pixels were unresponsive and are not propagated (Chantler *et al.*, 2015 ▸). The detector geometry is presented in Fig. 1 ▸. Simultaneous data collection in both modes is important because previous XERT (Chantler *et al.*, 2001 ▸) and hybrid (Chantler *et al.*, 2015 ▸) analyses have considered concentrated samples and this gives a critical comparison of fluorescence with transmission in a regime where both methods are competitive.

## 5. The self-absorption functional   

X-ray absorption for transmission measurement is given by the Beer–Lambert equation 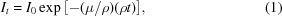
where *I*_0_ is the intensity of the incident X-ray beam, (μ/ρ) is the mass attenuation coefficient of the absorbing material, ρ is the density of the material and *t* is the thickness of the material. We cast the formula into a form where the quantities (μ/ρ) and (ρ*t*) are clearly measurable. For a well defined ordered solid (foil, copper, crystal plate) the characterization of the sample by mass *M* and cross-sectional area *A* defines an average *M*/*A* = (ρ*t*) integrated column density. Relative to this average, the local (beam spot) (ρ*t*) integrated column density can be (experimentally) well defined. On changes of temperature, the integrated column density is normally unchanged, whereas the density can change dramatically (a few % or more for solids). Conversely, we can measure the map of thickness *t* with a micrometre or profilometer but we will not know the density. Voids, bubbles and roughness would then yield a large error in the determination of *e.g.* the linear attenuation coefficient μ. Similarly, if we have a uniform solution, liquid or frozen, to first order the cell area may be constant and uniform including with temperature changes, whereas the density is not. The integrated column density (ρ*t*) remains characterizable, and (μ/ρ) is therefore measurable and an intrinsic property of the system like barns per atom. Theoretically, we can have predictions of the absorption and attenuation coefficients assuming a theoretical density. However, the core theoretical predictions of *FEFF*, *EXCURVE*, *FDMX*, *FFAST* *etc.* are in barns per atom, or in the case of the mass absorption coefficient (μ/ρ)_pe_ in cm^2^ g^−1^. The most direct comparisons with theory relate to (μ/ρ)_pe_ or (μ/ρ).

We use a derivation suitable for development and application to experimental data (*cf. e.g.* Pfalzer *et al.*, 1999 ▸). The equations within *SeAFFluX* are based on the (core) self-absorption functional,
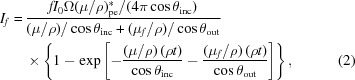
where *I*_0_ and ρ are as defined in equation (1), *f* is the fluorescence yield [usually for the specific fluorescence spectrum given by the region of interest (ROI), *e.g.* *K*α fluorescence], the asterisk indicates that only the component absorbed in the active centre producing a fluorescent photon is relevant, ‘pe’ signifies that only the photoelectric absorption coefficient is considered, Ω represents the solid angle subtended by each detector (pixel), θ_inc_ is the incident angle of the X-rays on the sample relative to the normal, θ_out_ is the angle of emission of the fluorescent radiation from the sample relative to the normal, (μ_*f*_/ρ) represents the mass attenuation coefficient of the material at the energy of the fluorescent photon, *t* represents the sample thickness and *I*_*f*_ is the total number of fluorescence photons produced in a small solid angle Ω centred on θ_out_ [*cf.* Chantler *et al.* (2012 ▸), Troger *et al.* (1992 ▸) and Booth & Bridges (2005 ▸)].

Since each detector pixel represents a different θ_out_ from the sample surface, there will be a different self-absorption functional for each pixel. This is not simply an overall scaling factor as some simpler models have used, but an energy-dependent functional since (μ/ρ) is a function of energy. With the self-absorption functional, we define and constrain the common parameters across all pixel measurements and remove this effect from our spectra, hopefully yielding data proportional to 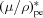, incorporating the results of Chantler *et al.* (2012 ▸) into the new software package *SeAFFluX* to correct for this systematic self-consistently. Chantler *et al.* (2012 ▸) did not attempt to remove the self-absorption; instead, they determined a corrected mean of the pixel spread, with a large component of self-absorption remaining in the fluorescent spectra.

In a standard XAS experimental setup, we have a good measure of the flux entering the system by using an ion-chamber detector at the start of the beamline. With careful attention to experimental details, we can then compare this flux with the intensity of radiation detected at the end of the beamline and correct for systematic losses. To correct for the upstream attenuation, we provide the following functional: 
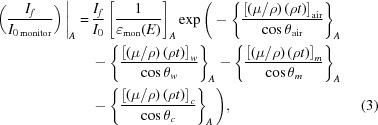
where *I*_*f*_ and *I*_0_ are as defined in equation (2), ∊ represents the overall quantum efficiency of the detectors, θ_air_ represents the incident angle of the radiation to the air (always perpendicular), *t*_air_/cos θ_air_ represents the path length that the photons take through air between the sample surface and the front face of the detector, and similarly for any window materials *w*, for the monitor gas path *m* and for the cryostat or other gas *c*. A common setup uses silicone adhesive on Kapton (polyimide) windows. The subscript *A* indicates that this equation describes absorption (transmission) components, in this case upstream of the sample photoabsorptive event. The (μ/ρ) terms represent the mass attenuation coefficient of each material and are functions of energy. The experimental thicknesses and densities were measured to a good accuracy (Chantler *et al.*, 2015 ▸).

Signal is lost due to attenuation through the air path, Kapton tape, detector windows and other materials in the experimental setup. If the X-ray photons pass through a certain thickness of any particular medium, then some fraction of them will be attenuated and not reach the detector.

A second functional is required to account for fluorescent photons emitted from the sample surface. In ‘transmission’ mode all experimental components are exposed to some fraction of the incident radiation from the synchrotron source. Thus, all experimental components will be exposed to the monochromatic source radiation, at whatever energy is being produced by the source. The experimental transmission setup is arranged in a linear fashion, and a significant amount of radiation is transmitted through the sample. Conversely, in ‘fluorescence’ mode all experimental components downstream from the sample, towards the fluorescence detector, are arranged perpendicular to the path of the incident source radiation (Fig. 3 ▸). Hence, components downstream from the sample are only exposed to radiation at specific fluorescence energies. This means that equation (3) applies to all components in transmission mode (including upstream and downstream components), and for upstream components similarly for both transmission and fluorescence. A second functional considering elements downstream of the sample, that are only exposed to fluorescence radiation, is given by
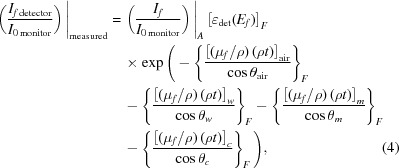
where the symbols remain the same as before. In addition, (μ_*f*_/ρ) represents the mass attenuation coefficient at the energy of the *K*α fluorescence photon and the subscript *F* indicates that this equation describes the components of fluorescence photon attenuation, *i.e.* downstream interactions towards an arbitrary fluorescence detector.

Equation (4) is not a function of energy [unlike equation (3) *per se*] but it is a function of geometry, source and angle, and hence of pixel or detector segment. In equation (3), all values for θ are constant (*ca* 0° or 45°, plus offsets accounting for experimental alignment). This is not the case for equation (4), since the downstream fluorescence detector has multiple pixels at different relative angular offsets. Often, the centre of the detector is aligned to be 90° relative to the path of the monochromatic source radiation. The different angular offset for each pixel results in a different path-length term, *t*/cos θ, and hence a different self-absorption factor for each pixel. There is a symmetry of response for pixels above and below the central line, but in general each pixel has a different correction factor. Variations in amplitude and spectral structure for a given pixel or solid-angle detector can arise from:

(i) [∊_det_(*E*)/∊_mon_(*E*)] overall detector efficiency for each element [equation (3)], including variations in the inelastic peak and shape with *e.g.* scattering angle and how well the detector and pixel definitions of the ROI are set up for each channel, including for example if the ROI values are set poorly enough to include significant but varying percentage contributions from the fraction of characteristic *K*β radiation, assuming that the ROI was set for the *K*α fluorescence.

(ii) A correction for dead time is assumed correct to a high level and without significant error, though if there are wide variations in count rate this would also affect the effective efficiency of a pixel unless corrected for in the usual manner (as done with this data set).

(iii) A subtlety relates to the ROIs cross-collecting *e.g.* some of the elastic scatter peak or the small Compton scattering shoulder, captured within the ROI window (which depends on the width of this peak, the angle and the ROI, and thus varies between pixel elements).

In principle, significant issues or problems of the above types should have been addressed by the experimental setup, pixel calibration, dead-time processing and ROI definitions well before data collection. In practice, all data sets have such limitations, but hopefully at a minor or negligible level. For the current data sets, the reference transmission data include all attenuation corrected for the very dominant matrix background (Chantler *et al.*, 2015 ▸), and the background attenuation from inactive orbitals and non-nickel atoms has been (roughly) subtracted for detailed comparison. We do not go into the details and limitations of such procedures here. For the fluorescence data, however, the tail below the edge is presumably not fluorescence photons because there is no significant photoelectric absorption coefficient below the edge to the continuum. There can of course be some excitation to an excited bound state for the pre-edge, which could relax with the production of some fluorescence but at a different energy, and the rest of the photoabsorption is to *L* or *M* shell electrons which will relax without production of a *K*α photon. Hence, the background signal is likely to be *e.g.* affected by the ROI and perhaps indicative of a small scattering shoulder accepted by the ROI [points (i) and (iii) above]. These should not be background-subtracted in the same manner as for transmission data because the cause and shapes are different. It has been claimed that they should be subtracted as per standard transmission spectra processing, either by average or for each individual pixel. This also is a poor approach, but a general empirical subtraction is of course possible. Some workers arbitrarily subtract a constant offset for this purpose, but if such a peak is *e.g.* the elastic peak, then a constant subtraction is not very appropriate.

Most older detectors and beamline collection stations define an ROI and hence cannot explicitly correct for this. Some newer full-spectrum analysis can fully address this by a complete mapping of the scattering functionals, but this is quite a laborious process and rarely performed. In our example, we do not subtract in advance but use the full set of data to investigate the functionality. Whereas in Fig. 1 ▸ this small background looks fairly flat and uniform, it does not actually need to be subtracted until one extracts the χ from the absorption coefficient spectra. Conversely perhaps, Fig. 4 ▸ emphasizes the significance of the different slopes above the edge: by normalizing to the white line and plotting lower ranges of energy, this enhances the apparent but not the real effect. The correct approach is to see the impact of any of these systematics on the *SeAFFluX*-corrected spectra, before processing to χ. What we will find is that the spectrum is well corrected for this variation and any such concern can be removed after inversion and processing for self-absorption. One might indeed argue that any photons, scattered or otherwise, will be subject to the same downstream energy self-absorption, though perhaps with slightly different energies and coefficients, so that it is no surprise that the software and method are effective here too. We want a robust solution which works effectively for most beamlines to high accuracy, and we demonstrate that herein.

(iv) The dominant effect after the pixel/amplification efficiency is the absorption and self-absorption corrections, especially including the detailed geometry.

## 6. Inversion in X-ray fluorescence analysis   

Since it is 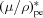 that we ultimately want to extract from our data, we recast the previous formulae to reflect this inversion. To extract 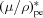 or χ from a fluorescence measurement, one must invert the above equations, and we look at the final observed measured 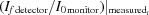 for each pixel *i* from equations (2), (3) and (4): 
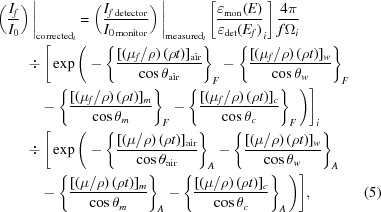


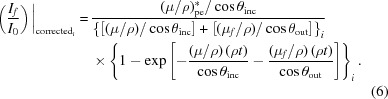



Most of the terms in equation (5) are defined or measured and are smooth background functions of energy. The detector efficiencies are complex and pixel-dependent and are retained as an array of fitting coefficients. Potential challenges lie in equation (6) (Booth & Bridges, 2005 ▸), because 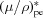 appears as stated, but is also included in 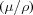 = 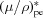 + 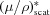 + 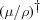 in the denominator and in the exponential, where * refers to the active electrons in the active orbital and 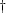 refers to all other atoms (matrix, solvent *etc.*) and orbitals. Only the (mass) photoabsorption coefficient for the electrons in the active orbital can lead to fluorescence. Equation (6) is noted to be formally not invertible. Indeed, in a pure mon­atomic sample like a metal foil, where 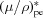 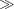 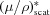 + 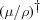 + 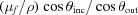, the XAFS oscillations can be (almost) completely damped to zero, in which case there is no signature for the XAFS oscillations or for χ, at which point the equations are indeed formally not invertible. In such cases there is no XAFS. However, in almost all real cases the equation is invertible.

Here, we are deriving and correcting 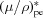 and not directly χ (Booth & Bridges, 2005 ▸) for three reasons. Firstly, measurables from theory relate to 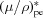. Secondly, we may wish to recover signal just below the edge. Thirdly, different analyses and expressions use the edge step or a spline or an atomic like spectrum in the denominator for χ and we wish to avoid those concerns here, which would change the scaling and the error correction. However, one convenience of the χ prescription is that it normalizes for a scaling error implicitly. For the general case we expand equation (2) (see Appendix *A* [App appa]): 
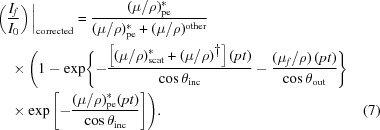



For faster convergence of the expansion, we can recast this in terms of the baseline, atomic or non-oscillatory XAFS 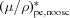 = 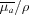, with 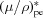 = 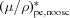 + 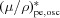 = 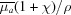, where the oscillatory components are given as 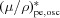 = 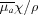. Then (see Appendix *A* [App appa]) the expansion can be phrased around
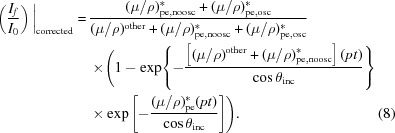
Incidentally, in units based on Booth & Bridges (2005 ▸) this can be represented as 
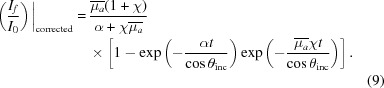
The pixel-dependent dispersion is seen by all θ_out_ terms in all equations, and especially in (μ/ρ)^other^ and α in the above equations. The so-called self-absorption is given by the (μ_*f*_/ρ) terms. Effective inversion requires all terms of all equations.

The details of our inversion methodology are presented in Appendix *A* [App appa]. We recast equation (7) in terms of 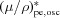 = 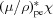 and outline the second- and third-order Taylor expansions around 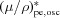 = 0. We make the simplifying approximation to separate out the exponential in equation (7) and divide through by this term as a second step. As indicated in Fig. 6, this does not introduce significant error into our result. Important advantages are that the inversion is presented in terms of the full 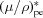 coefficient, or the oscillatory component of the coefficient; that it links directly to theoretical coefficients; and that the inversion is for the first time directly comparable with the transmission raw data. It also enables direct extraction of χ but without a range of limiting assumptions.

## 7. Self-absorption modelling: scales and logic for a particular example   

The application of equation (3) to fluorescence data produces an energy-dependent functional in the detected spectra of 33 spectra (in our test examples), all bunched together at a reference point on the left, which then ‘fan out’ at higher energies due to their different gradients arising from equations (5) and (6). We consider the location of Bragg glitches (monochromator three-beam diffraction locations for the monochromator crystal) and remove them accordingly in the initial processing (Appendix *B* [App appb]). A simple processing normalization is illustrated in Appendix *C* [App appc].

Consider the dispersion of the pixel responses, even after normalization, across an extended range of energy, as seen in Fig. 4 ▸. The theory implemented in the *SeAFFluX* package is based on equations (2), (3) and (4). The angle of incidence on the square-planar detector, θ_out_, can be separated into horizontal and vertical components relating to the location of each detector pixel individually. The horizontal component is given by θ_out, *h*_ = 45° + tan^−1^(*nC*/*L*), where *n* is the number of horizontal pixels from the plane of incidence, *C* is the distance between pixels and *L* is the separation between sample and detector. Note also that a small offset from 45° may be necessary due to misalignment. The angle is given by cosθ_out_ = cosθ_out, *h*_cosθ_out, *v*_. Therefore, the vertical component is given by θ_out, *v*_ = tan^−1^(*mC*/*L*), where *m* is the number of pixels from the vertical plane of incidence. Equation (3) was extended from Chantler *et al.* (2012 ▸).

Fig. 5 ▸ illustrates the predicted effect of self-absorption and attenuation on the detected fluorescence spectra using a simple simulation of a reference nickel spectra from the *FFAST* tabulation (Chantler, 1995 ▸) as detected by six horizontally aligned detector pixels, as opposed to the simplified method presented in Appendix *C* [App appc]. The theory here predicts the rising trend with energy and the increasing dispersion between pixels at higher energies. This is a significant proof of concept. We can therefore proceed to invert the self-absorption and attenuation systematic using *SeAFFluX*. This must include all of the above equations, or it may be neither predictive nor invertible.

Each pixel spectrum in the fluorescence detector has been fitted with an independent overall scaling factor. Thus, the final spectra are not absolute but presented on a relative scale, unless the spectrum is calibrated independently, for example using *XERT*. In addition to these overall scaling factors, two small offsets are fitted to account for potential misalignment between the fluorescence detector and the incident fluorescence beam (nominally at 45° in the horizontal and 0° in the vertical). All other parameters are given by experimentally measured distances/thicknesses, with uncertainties. These additional coefficients (air path, window and other thicknesses, *etc.*) could be fitted. However, we find that the measurements appear quite accurate within their uncertainties, so we can proceed accordingly.

The *y*-axis scale in the reduced data becomes either a scaled 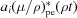 or 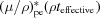 (Figs. 4 ▸ and 11). Because of the relative efficiencies of the detectors and any uncertainty in the solid angle and sample depth, this scale cannot have the same meaning as in transmission measurement. The set ROI (upper and lower level discriminators), which is typical of most fluorescence measurements at most XAS beamlines and other applications, ideally excludes any matrix absorption and scattering, any elastic scattering peaks, any non-active atoms in the molecule or solid and any background attenuation from other orbitals. So in the one-particle approximation (neglecting shake *etc.*), the measured fluorescence signal is 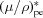. Representing *I*_*f*_/*I*_0_, the scale must be dimensionless, just like the logarithm in the transmission measurement. This contrasts with transmission measurement which includes all attenuation processes from active orbitals, background orbitals, inactive atoms in the molecule or solid and any matrix (μ/ρ) – but of course only measures the signal in the forward (and backward) directions.

In transmission, the value of (μ/ρ) can be determined on an absolute scale directly interpretable from the full integrated column density (ρ*t*) of the sample. In fluorescence, at best this relates to some effective depth and so, while hopefully linear and proportional, it is a scaled relative measurement. Hence fluorescence measurements in general can measure XAFS but cannot (directly) measure absorption or attenuation coefficients on an absolute basis. As a rough approximation, one might interpret *t*_effective_ to correspond to a 1/*e* depth, which will be energy-dependent but roughly corresponds to 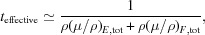
corresponding to the total attenuation of the incident and fluorescent fluxes. This is certainly a functional of pixel angle.

Table 2 ▸ provides the scales relating to the inversion procedure dealing with equations (6) or (8) for our test cases, complementary with Table 1 ▸ for the overall experimental variables and the inversion and modelling relating to equation (5). The columns illustrate energies at the fluorescence *K*α, at an energy just below the edge, at an energy around the white line, at the strongest peak of the data, at an intermediate value of *k* and at a high-energy limit, to show the spread and trend of the slopes. The first rows show the contributions of the solvent and the nickel complex and the active Ni orbitals to the total sample attenuation. The next section illustrates the rough scale of the dispersion, both between pixels and angles and across energy for the experimental data, which should of course be consistent with the theoretical modelling. The next three rows present the variation in the mass attenuation coefficient which is being addressed, and the last row gives a scale of the χ across the spectrum, above the edge of course. It is important to recognize that the effects addressed in this example are large and significant, and need to be corrected for to obtain reliable absorption data with which to compare theory.

There are two key potential singularities in equation (8): the presence of the oscillation in the denominator damping the observed oscillation in the numerator, and the presence of the oscillation in the exponential which dampens the effect of the denominator but has been largely neglected in the application of the theory. In Fig. 6 ▸ we compare the significance of the influence of the denominator on the inversion using equations (7) or (8): 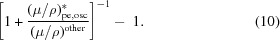
Note that the denominator (μ/ρ)^other^ [or 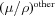 + 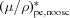] is always positive, but 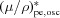 must oscillate and so is generally positive and negative; in the illustration of convergence, this is also defined by the accuracy or the (slight over-)estimate of 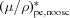. This scale of the ratio above the edge (*e.g.* *k* = 0) (the fractional correction) is 1% for the white line, has a background level of 0.4% and shows oscillations of 0.2–0.3% for the first peaks of the XANES/XAFS spectrum, declining rapidly to below 0.1% for the main XAFS region and beyond. Because the ratio is well behaved, the Taylor series expansion converges very quickly (Figs. 7 ▸ and 8 ▸). Although the real spectrum will have an XAFS oscillatory component of approximately zero at high energies, the software will be more or less robust in extracting this, but it will have no significant effect on the relative magnitude of oscillatory corrections in the result. As in Appendix *A*, we use the exact solution of the expanded inverted denominator to first and second order *etc.*, but really first order is an excellent approximation.

The first-order Taylor series expansion of the denominator yields a quadratic, which is easy to solve and almost a full correction for all pixels (in this case). This is proven by the expansion to second order in the denominator, which yields a cubic equation. The convergence appears complete. In the presence of noise it is crucial to ensure that the experimental data are normalized to the correct background level, and that the scale of oscillations in the numerator therefore reflects the scale of oscillations in the denominator.

To date, the other term in the inversion formula has not been invertible and has usually been ignored. This is the exponential. Remember that we have already corrected for all upstream and downstream optical components and here only consider the sample with matrix. In our test case, the matrix is dominant so that the corrections are large and pixel dependent. We examine the percentage error in the scaling with the following expression [following equation (8)]: 
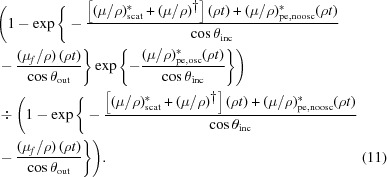
The exponential is not heavily affected by this because the sample is ‘thick’. Indeed, although the path-length differences are large, and the θ_out_ variation is large, the effect on the inversion and on the ratio is less than 1% everywhere (Fig. 9 ▸), including a 0.4%–0.8% background-level shift, a similar white-line-level shift, and a relative shift of even the first few XANES peaks of the order of 0.1%. Hence, this is both directly invertible and has a minor effect on the relative scaling of XAFS oscillations. The effect of the product of these corrections is small (Fig. 10 ▸) and the inversion is well defined and directly inverted, with an uncertainty less than 1% overall for any pixel and actually much better than that if we are considering a possible impact upon χ.

For the system considered here, the neglect of the denominator or exponential coefficients for the oscillatory components in our inversion process introduced an error of 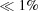. However, the full inversion formulae have a great advantage for high-*Z* inorganics and metals, for example. The methodology is useful in general and makes a very significant contribution to the investigation of different experimental systems. Indeed, it has a very large effect on our spectra, but in our case the fan of pixel spectra is due both to the self-absorption and also the downstream absorption, with different paths and angles to the pixels. We have emphasized these in our equations and inversion formulae.

## 8. Results of spectral correction on dispersion and variance of experimental data   

This approach (Fig. 11 ▸) represents a dramatic improvement over the previous ‘normalizing’ model, and also over earlier approaches. The dispersion between pixels is almost eliminated for both data sets, indicating that variations in slope and divergence with energy are caused by attenuation and self-absorption. The minimal fitting coefficients imply that the measured parameters are sufficiently accurate and physically meaningful. The high-energy dispersion seen in the previous ‘normalizing’ model has been fully corrected.

The functional with energy now looks exactly like that for transmission, with the gradient above the edge decreasing just as for theoretical photoabsorption. Below the edge, the value is flat and almost zero, as expected from the setup of the ROI, so the ROI excludes most other fluorescence and scattering processes. Any residual scattering background can be removed following the ‘pre-edge background subtraction’ used in most XAS analysis, but noting that the specific functional form may not be that from the inactive orbitals or elements as per transmission background subtraction, and may not be a constant as used in some software processing for fluorescence data. Incidentally, the matrix contributions to the transmission signal were quite large in this experimental example but were subtracted accurately by careful measurement (Chantler *et al.*, 2015 ▸). Both transmission and fluorescence measurements were taken simultaneously on the same samples.

**Figure 2:**
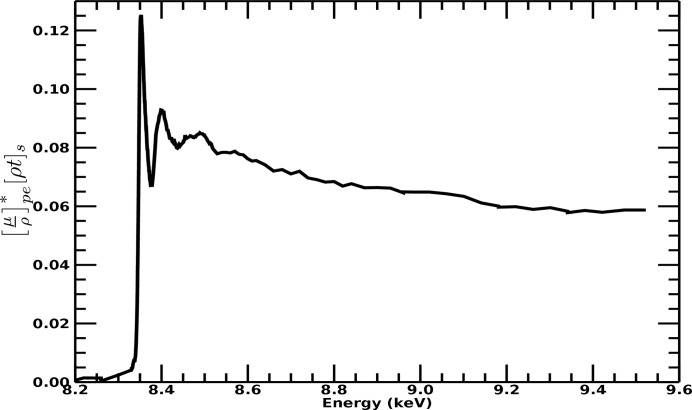
Published transmission spectra for the active Ni in the Ni *i*-pr complex, on an absolute scale, displaying the expected XAS spectral trend for absorption coefficients (Chantler *et al.*, 2015 ▸). These measurements were taken simultaneously on the same samples as Fig. 1 ▸. This illustrates the large differences in structure and spectral profiles of transmission data (μ/ρ)(ρ*t*)_*s*_ from the fluorescence measurement, 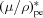 shown in Fig. 1 ▸, observed in many data sets. The standard error across the spectrum is of the order of 10^−4^ in absolute units or <0.01% on the raw data.

**Figure 3:**
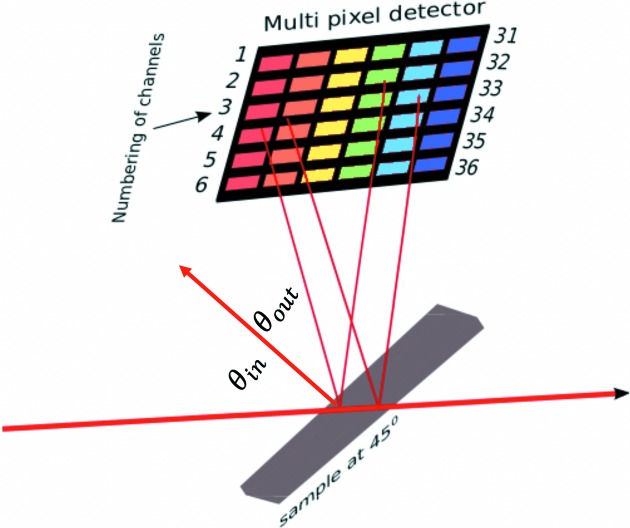
A schematic diagram of the fluorescence detector geometry.

**Figure 4:**
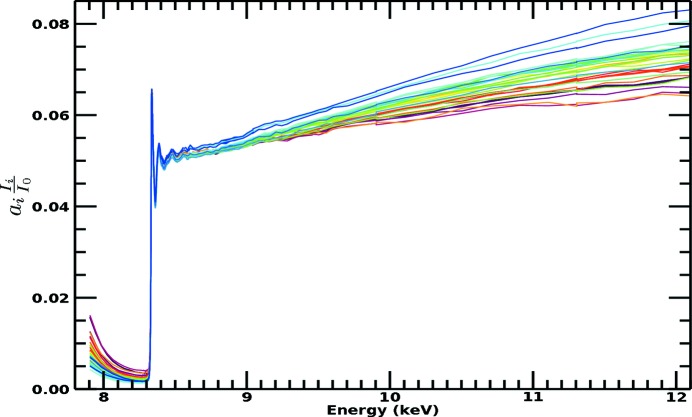
Fluorescence spectra for the nickel *i*-pr complex extended to higher energy. The increasing dispersion with energy is particularly evident across the larger energy range. Detector efficiency normalization is not sufficient to remove the large self-absorption signature and systematic from the spectra. An overall scale factor *a_i_* is intrinsic to the fluorescence data because of the pixel efficiency, as discussed in the text. The colour scheme is consistent with previous plots.

**Figure 5:**
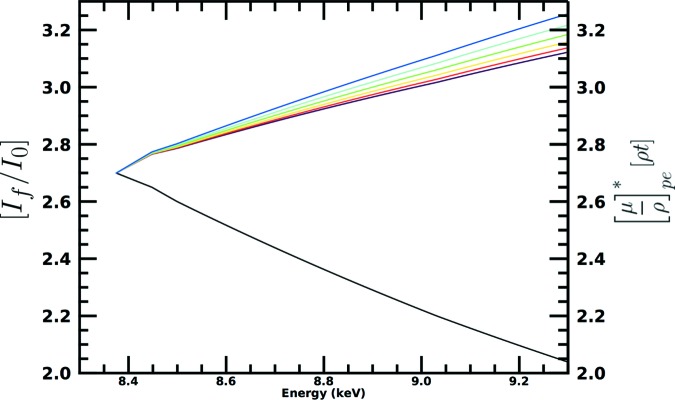
A simple simulation of a nickel metal X-ray absorption spectrum (shown in black, axis on right) transformed by self-absorption and attenuation (colours matching pixels, scaled axis on left), using the same distances and geometry as in the experimental data considered here. Oscillatory XAFS is not simulated in this figure.

**Figure 6:**
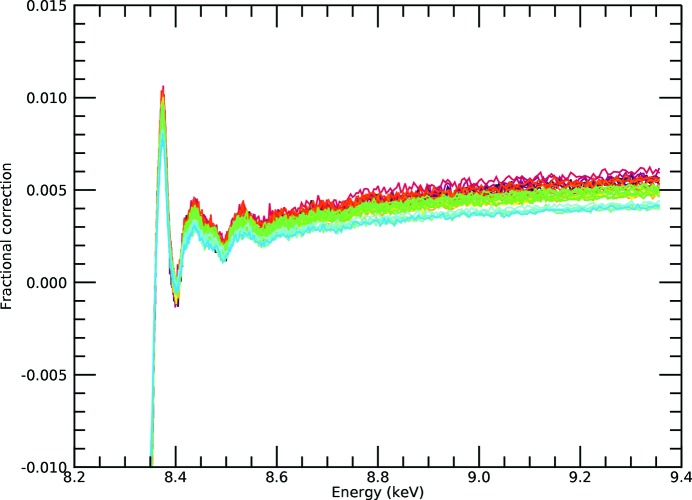
For the *i*-pr data set, the fractional correction from the denominator term in our test case is particularly small, even before Taylor expansion of the 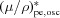 term. The fractional correction is as given in expression (10), as a function of energy and after inversion. Therefore, we expect the inversion to be relatively straightforward and to converge quickly and successfully, even though the exact solution of cubics and quartics can be challenging for mathematical software in the presence of noise.

**Figure 7:**
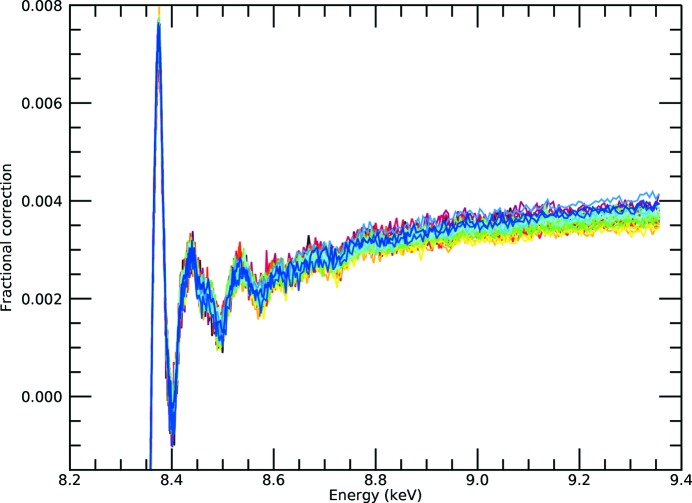
Fractional correction after first-order Taylor expansion correction [equation (8)] for the *i*-pr data set. The base level is about 0.4%, the white line peak is about 0.8% and the oscillations of the first few peaks are of the order of 0.2–0.3%, just as expected from the ratio. The inversion appears robust for all pixels to below a 0.1% level.

**Figure 9:**
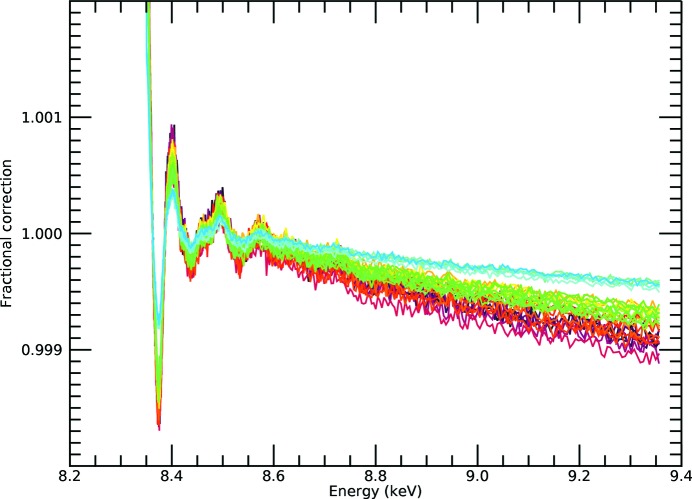
For the *i*-pr data set, the effect of the 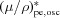 term on the exponential component of the inversion formulae is ∼0.2% at the edge and 0.05% in the oscillations and background [equation (8)].

**Figure 10:**
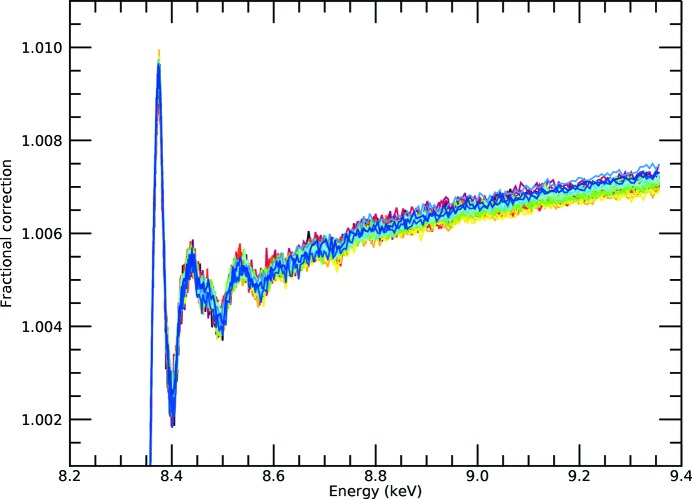
The combined effect of the second-order expansion of the expression (10) correction multiplied by the correction in expression (11) for the *i*-pr data set. Both corrections are small and well converged and the exact form of the exponential is clear, yet the neglect of the oscillatory component introduces a very minor change overall.

**Figure 11:**
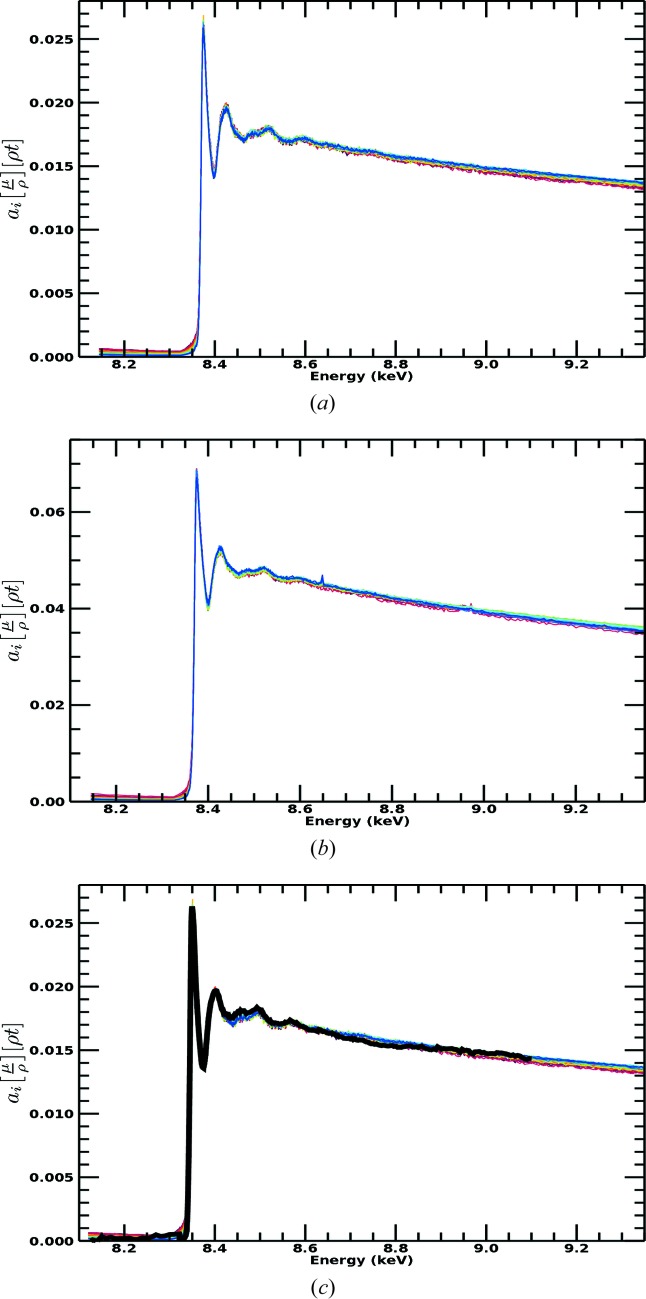
(*a*) Ni *i*-pr *SeAFFluX*-corrected spectra. (*b*) Ni *n*-pr *SeAFFluX*-corrected spectra. (*c*) Panel (*a*) with a scaled overplot of the transmission XAS spectra. A dramatic reduction in dispersion is observed in the (*SeAFFluX*-) corrected spectra. Crucially, the fluorescence spectra (all pixels are plotted) now display the correct decreasing trend at higher energies consistent with the absorption data in Fig. 2 ▸, as illustrated in Fig. 11 ▸(*c*). The fluorescence scale has one free parameter *a_i_* corresponding to the pixel efficiency normalization.

Remarkably, we can superimpose the spectrum and detailed structure of the oscillations from transmission over that of the fluorescence corrected spectra, Fig. 11 ▸(*c*), without any calibration from one to the other, and the overlap is almost perfect. The magnitude and overall trend with energy are now in excellent agreement with the absorption data in Fig. 2 ▸. After correction for the dominant systematics, the dispersion is greatly reduced and the spectral shape follows the absorption coefficient trend. This is a powerful demonstration of the accuracy of the attenuation and self-absorption application, and proves that such data can be used in XAS analysis as for high-accuracy transmission data. We do *not* use a simplified ‘thin limit’ or ‘thick dilute limit’ formula for fluorescence to impute validity of analysis (Chantler *et al.*, 2012 ▸; Newville, 2004 ▸; Bunker, 2010 ▸). The effects we have presented and observed, and which are almost universal in fluorescence data collection (dispersion of pixel slopes with angle and energy, and trending upward slopes), require all of equations (2), (3) and (4). The thin sample limit has no rising slope nor any dispersion between detector pixels: if you see a rising slope, or dispersion between pixels, the thin sample limit is not adequate or not sufficient. Any fluorescence data with any dispersion between pixels, or any flat or rising slope above the edge, implies that additional correction and equations are required to explain the data and invert the attenuation effects.

**Figure 12:**
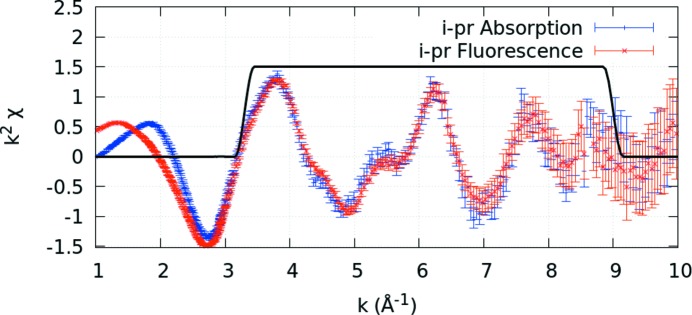
Transmission versus fluorescence spectra. An excellent agreement is found between the two spectra within the Hanning window, indicative of the success of our self-absorption correction methodology. Some discrepancies remain between the two spectra, particularly in the application of background subtraction, edge removal and spline removal, an observation made possible by our propagation of statistically robust experimental uncertainties. A subsequent publication will present this in detail and contrast the results of structural modelling of these two data sets.

The formulae herein allow the recovery of structure with the correct magnitudes. Fig. 11 ▸ demonstrates excellent agreement in processing for two independent data sets. Fig. 12 ▸ confirms that this agreement is consistent when the spectra are converted to χ: the angles are identical, the fluorescence depths are identical, and the concentrations of matrix and active species are almost identical. The 1/*e* depths depend upon the whole sample, especially including the matrix, and not just the absorber of the active edge. In other studies, the apparent scale can be significantly influenced by the definition of a reference pixel channel for normalization and the relevant vertical and horizontal angles of that offset. The uncertainties in the figure are not explained here and will be discussed further elsewhere.

## 9. Discussion: theoretical and analytical insight   

Booth & Bridges (2005 ▸) wrote a landmark paper, and they avoided the thick sample limit and provided functionals for χ. However, we have provided functionals directly correcting for the mass absorption coefficient, the scaled equivalent or the absorption coefficient for the active species, and have provided software for the same.

Booth and Bridges also discussed potential inversion of their (final) formula for χ, which looks very little like ours. Their inversion uses 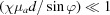. To invert the equation in general demands consideration of the remainder of the optics, as we have discussed. To correct for absorptive effects, one must consider them when they are potentially large, and one should be able to prove this in comparison with the raw or minimally processed data, without *e.g.* distortion from splines, or denominators of the χ transform being the edge-step height rather than the theoretical atomic absorption above edge background. We provide alternative formulae and Taylor series expansions with exact solutions to second and third order which converge, and we confirm and check these compared with the ratio arising from the denominator and from the exponential. In this way, we demonstrate the magnitude in any data sets and the approach to convergence. Hence, if perhaps the data are near a non-invertible singularity (*i.e.* where no XAFS oscillations are measured), this will be flagged in the analysis.

We have investigated a significant limit of large attenuation and self-absorptive effects, especially towards the limit of large solid angle and significant change of attenuating and self-absorptive effects between pixels. The success of the approach is proven by the causal nature of the enormous pixel variation, and by the fact that the spectra compress almost perfectly by applying the corrections explained by the equations above. If we had ignored some key component of the absorptive correction, then the pixels would not recover the same form and shape, and would not recover the shape of the absorption curve. In other words, neglect of key angle-dependent and energy-dependent components of the optics would make the equations non-invertible.

A reader from a biological background may be concerned that this data set is ‘quite concentrated’ (15 m*M* or 0.1%) compared with some fragile and dilute species, and (yet) has increasing noise above *k* = 12. The data were collected on a bending-magnet beamline, whereas current advanced beamlines can easily have 100× the flux, count and statistics. Much lower concentrations are quite feasible with hybrid techniques; indeed, we get promising results for 1.5 m*M* data in this way, even at a bending-magnet beamline. Ergo, the community should be encouraged to look at data even of dilute systems beyond the XANES region: it is completely feasible to have high accuracies on dilute data fully across the XAFS region in both transmission and fluorescence at low concentrations, with limits yet to be determined.

## 10. Conclusions   

This work demonstrates that structural information can be extracted from fluorescence spectra of at least the same quality as transmission data, and both methods are fully valid for 15 m*M* solutions or 0.1% *w*/*w* samples. This can apply to samples of interest not well suited to transmission experiments. The methodology presented in Section 6 is readily adaptable to arbitrary samples of interest; different approximations can be appropriate under different experimental conditions. The results presented here are far from the limit of what can be achieved with this technique, and we emphasize the general applicability of this methodology. The example considered and analysed in this paper is fairly dilute, towards the ‘thick dilute limit’, but the same logic would equally apply to the analysis of fluorescence spectra collected on a thick metal foil, a sample with a support, or a thinner or more dilute sample. Equally, the methodology presented here applies to 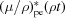 or 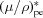 and can thereby be applied directly to absorption-edge distortions and, according to the user’s preference, can be pre-edge subtracted and converted to a functional for χ, as demonstrated here.

We have presented theory and software for processing multi-pixel fluorescence data for XAS and other techniques. We have developed the theory and application of the correction (inversion) of these systematics and of the inversion formulae for self-absorption, and have applied them to test cases with large matrix, upstream and downstream contributions. This approach and theory are easily valid for systems containing up to 1.5 m*M* or 100 p.p.m. of the active absorber, but we expect much more with refinement of the approach. Further work will explore this challenge in greater detail, explain the estimation of uncertainty from such data, and investigate structural modelling of this self-absorption corrected data.

The software is available for download with this publication as supporting information (Appendix *D* [App appd]).

## Supplementary Material

SeAFFluX software and manual (zipped). DOI: 10.1107/S2052252519005128/hf5937sup1.zip

XAFS structural files in efeffit and cif formats (zipped). DOI: 10.1107/S2052252519005128/hf5937sup2.zip

## Figures and Tables

**Figure 8:**
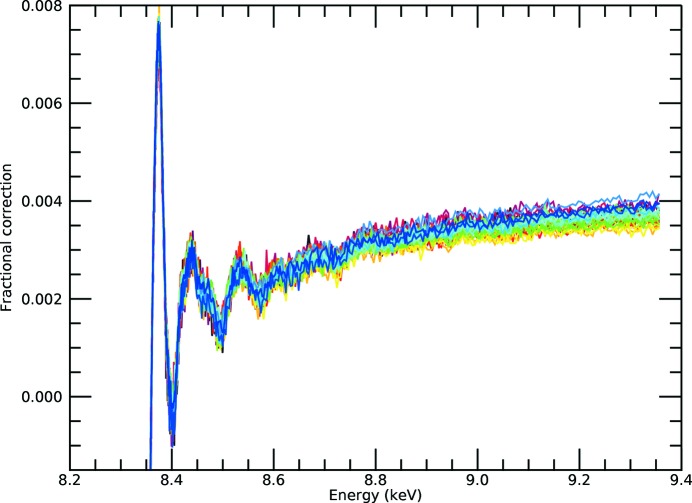
Fractional correction after second-order Taylor expansion correction [equation (8)] for the *i*-pr data set. This is very similar to the result of the first-order Taylor expansion in Fig. 7 ▸, and shows that the correction is now well converged. The difference from the previous figure is not easily visible by eye – the data have converged.

**Figure 13:**
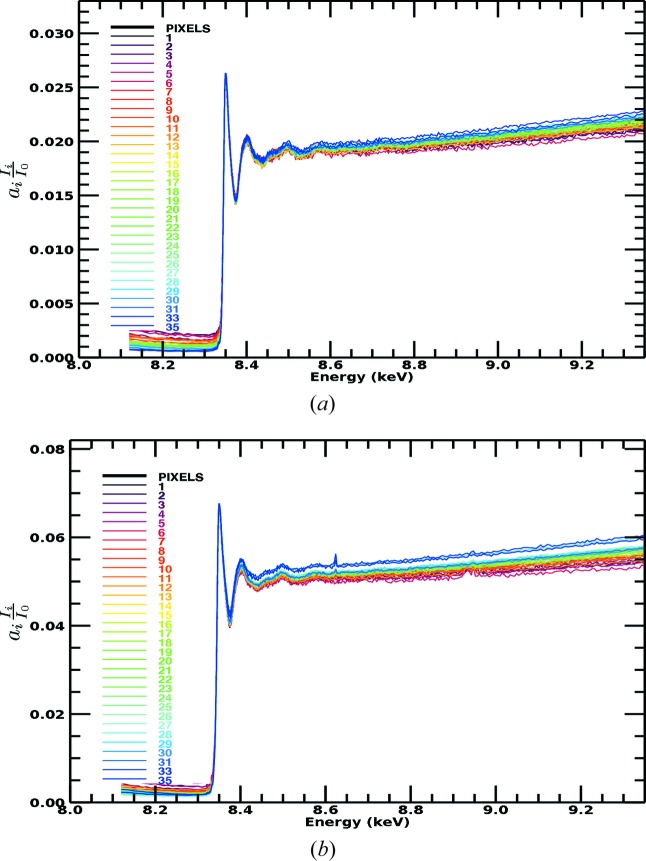
(*a*) The *i*-pr spectra normalized for detector pixel efficiency. (*b*) The *n*-pr spectra normalized for detector pixel efficiency. Normalization for pixel efficiency [∊_det_(*E*)/∊_mon_(*E*)] makes a significant improvement in the dispersion between spectra. However, significant dispersion and variance remain in the pre-edge region and increase at higher energies. Furthermore, the spectral trend with energy is not in agreement with the transmission data (Fig. 2 ▸). Typical applications of this limited approach pin spectra at the edge or pre-edge and diverge away from this empirical pin. This methodology is not sufficient for high-accuracy data analysis.

**Table 1:** Estimated attenuation contributions by the background absorbers using *FFAST* theoretical data of X-ray mass attenuation coefficients, and the geometry of the experimental setup For fluorescence geometry one needs to separate the incident flux at incident energy through the upstream path lengths, from the fluorescent emission path lengths, hence the parameters displayed in the table. The path lengths were determined from the measured geometry of the experimental set-up.

Background absorber	Chemical formula	Density (g cm^−3^)	Path length (cm)	(ρ*t*)_nominal_ (g cm^−2^)	(μ/ρ)_*FFAST*_ (cm^2^ g^−1^)	(μ/ρ)(ρ*t*)_*FFAST*_
Upstream, incident *E*, *e.g.* 8.45 keV						
Air	N_2_ (78%) + O_2_ (21%) + Ar (0.93%)	0.0011	28.9 (6)	0.03179	7.696	0.2447
Detector gas	N_2_	0.0012	19.0 (1)	0.02280	6.171	0.1407
Kapton (polymide)	C_12_H_10_N_2_O_5_	1.42	0.010 (1)	0.0142	5.610	0.0797
Silicone (adhesive)	CH_3_—Si_2_O_2_—C_4_H_9_	0.968	0.0060 (6)	0.00581	22.29	0.1295
He gas (cryostat)	He	0.0001785	1.1 (1)	0.000196	0.2633	0.0000516
Downstream, fluorescent *E*, Ni *K*α 7.39 keV						
Air	N_2_ (78%) + O_2_ (21%) + Ar (0.93%)	0.0011	8.2 (1)	0.00902	11.47	0.1035
Kapton (polymide)	C_12_H_10_N_2_O_5_	1.42	0.0060 (6)	0.00852	8.305	0.0708
Silicone (adhesive)	CH_3_—Si_2_O_2_—C_4_H_9_	0.968	0.0060 (6)	0.00581	32.59	0.1893
He gas (cryostat)	He	0.0001785	1.1 (1)	0.000196	0.2932	0.0000575

**Table 2:** Rough scales of attenuation in the 15 m*M* *n*-pr data set

Scale of attenuation or absorption	*K*α, 7.39 keV	Below edge, 8.0 keV	White line, 8.3 keV	XAFS, 9.0 keV	High *E*, 12 keV
(μ/ρ)(ρ*t*)_*s*_, sample = solvent + Ni	3.3	2.7	2.49	1.89	0.76
Solvent (μ/ρ)(ρ*t*)_*s*_	3.3	2.7	2.38	1.80	0.72
Ni (μ/ρ)(ρ*t*)_*s*_	0.02	0.02	0.11	0.09	0.04
Ni *K* shell 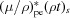	0	0	0.09	0.08	0.03
Fluorescence pixel variation *I*_*f*_/*I*_0_	0	0.001	0.018	0.019	
Pixel variation, % of fluorescence signal *I*_*f*, *i*_/*I*_*f*, *j*_	0%	200%	220%	300%	
Normalized with respect to white line *I*_*f*, *i*_/*I*_*f*, *j*_	0%	400%	1%	8–10%	25%
Increase from edge step (slope) *I*_*f*_/*I*_*f*, edge_			0%	12–20%	70%
(μ/ρ)_*s*_ ≃ (μ/ρ)_pe_, Ni complex, cm^2^ g^−1^	14	11	54	44	18
(μ/ρ)_scat_, Ni complex, cm^2^ g^−1^	0.65	0.62	0.59	0.56	0.44
Ni *K* shell 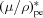 , complex, cm^2^ g^−1^	0	0	47	38	16
Maximum χ	0	0	0.5	0.01	0

## Appendix A Self-absorption modelling

### A1. Derivation of inversion of self-absorption functional

Previous work considered the ‘thin sample limit’ (Newville, 2004 ▸; Bunker, 2010 ▸) where the attenuation through the whole sample at the given angles is small and

Equation (6) then becomes

This ‘thin sample limit’ has no rising slope and no dispersion between pixels, so in general this limit does not apply to any real fluorescence data. Note, however, that for particularly thin samples the (pixel or energy) dispersion can be dominated by equations (3), (4) and (5) rather than (6). The corresponding ‘thick sample limit’ (Troger *et al.*, 1992 ▸; Newville, 2004 ▸; Bunker, 2010 ▸) assumes that the exponential goes to zero through the thickness of the material, so that there can be no directly comparable transmission measurement and equation (6) becomes

If  ≪  +  + , the sample is dilute and dominated by the matrix, solvent *etc.* (the ‘thick dilute sample limit’), and the XAS step and oscillations are scarcely damped by the  in the denominator. In general, the XAFS oscillations seen in transmission are damped in fluorescence. In this thick dilute sample limit, the XAFS oscillations are not dominant in the denominator and the equation becomes invertible, if necessary, with

### A2. Inversion of equation (7)

Equation (7),

where

can be expanded by applying a Taylor series expansion around :

This expansion will be appropriate for  < 0.2, or typically  < 0.14 and/or  < 0.2 with an error of the order of approximately 2–4% for this first-order expansion. Higher-order expansions have a larger circle of convergence.

Substituting back in,

Rewriting in simpler notation,

Then solve for *x* = .

#### A2.1. Quadratic approximation, first-order expansion of denominator only

or

Then solve for *x* = .

#### A2.2. Cubic approximation, second-order expansion of denominator only, ‘thick’ limit expansion

or

Then solve for *x* = .

#### A2.3. Cubic approximation, first-order expansion of denominator and exponential, ‘non-thick’ limit expansion

or

Then solve for *x* = .

### A3. Inversion of equation (8)

#### A3.1. Re-casting in terms of an expansion of

Note

Define

Taylor expansion around *x* = 0 yields

Rewriting in simpler notation,

Equivalently, in a cubic expansion:

The recommended order of operations for this inversion (and the default mode in our software) is to begin by solving equation (38) [cubic solution after expansion in terms of ]. This is then rescaled by the exponential term, also using expansion in terms of . After this correction, the data can be expressed in the most convenient form [most likely ]. The fractional corrections for each of these steps are illustrated in Section 7, as applied to our data.

This expansion will be appropriate for  < 0.2 and/or  < 0.2 with an error of the order of approximately 2–4% for this lowest order expansion. However, it is possible to approach limits of  < 1 and/or  < 1 where the correction is barely convergent for higher orders of expansion, as discussed.

## Appendix B Identifying Bragg peaks following Section 7

We highlight two features in Fig. 13 ▸ at around 8.65 and 8.95 keV. These monochromator glitches can also be seen in the ‘raw’ spectra in Fig. 1 ▸. These features are Bragg peaks, which arise when multiple sets of Bragg planes within the monochromator crystal are able to diffract X-rays of the same energy, leading to a decrease or increase in the diffracted intensity downstream towards the experimental sample (Chantler *et al.*, 2010 ▸; Chantler & Deslattes, 1995 ▸; Quintana & Hart, 1995 ▸; Sutter *et al.*, 2016 ▸; Bridges *et al.*, 1991 ▸; Li *et al.*, 1994 ▸). This effect can be seen in both sample measurements, although less clearly in the *i*-pr spectra.

Monochromator glitches are due to multiple-beam diffraction in the monochromator, in turn due to the azimuthal orientation of the monochromator crystal(s). Whereas a well formed transmission experiment should use matched detectors and this effect should cancel, the energy-dependent efficiencies of the upstream and downstream detectors, especially in fluorescence, leave this uncorrected. It is common practice to delete such channels, preferably after confirmation of their origin. We comment that a more advanced approach is to map them for each monochromator system and use this directly in processing; this can be a significant limitation to continuing in fluorescence measurement compared with transmission measurement. One can have other Bragg–Laue peaks or hollows from the sample, matrix or substrate diffraction, which of course will not cancel in either transmission or fluorescence measurement.

## Appendix C Normalization for detector efficiency, following Section 7

Each pixel in the Ge fluorescence detector has a different electronic characterization and possible variations in thickness, different active depth and dead layers, different electronic preamplification, and ergo different efficiency [∊_det_(*E*)/∊_mon_(*E*)]. Hence, the dispersion and variance seen across the spectra in Fig. 1 ▸ are significantly reduced by normalizing each pixel spectrum.

The results presented in Fig. 13 ▸ from pinning the normalization to a reference channel of energy show significant improvement. The oscillations above the absorption edge are much more clearly defined. However, the spectra remain inconsistent with significant dispersion in the pre-edge region, increasing at higher energies. The colours of individual spectra follow the pixel order and hence the position upstream or downstream. Clearly, the pixel position has a major pixel-dependent effect which is a function of energy, and the dispersion is not constant along the energy range. This behaviour is predicted by the θ_out_ term in equation (2), and is more significant at greater energies (Fig. 4 ▸).

Furthermore, and perhaps more significantly, the forms of the spectra do not agree at all, even qualitatively, with the classic absorption coefficient trend of a decaying intensity with higher energy after the absorption edge (Fig. 2 ▸), and so they cannot represent the attenuation coefficient to better than a 20–50% approximation above the edge. Ergo, analysis requires tools similar to those presented in this manuscript and software.

## Appendix D Energy uncertainty in tabulated data, following Section 10

We include as supporting information two data sets that have been processed with *SeAFFluX*. These data sets include uncertainties on the energy points, which have been interpolated from the previously published transmission data. A small energy offset was required in order to correctly align the two data sets. This offset was 1.50 eV for *i*-pr and 0.25 eV for *n*-pr. We also include the *SeAFFluX* code and installation instructions.
