# Supplementary material for: Solving self-absorption in fluorescence
Source: IUCrJ. 2019 May 10;6(Pt 4):586–602. doi: 10.1107/S2052252519005128 (PMC6608621; doi:10.1107/S2052252519005128)
Supplement: Supplementary file 1 [file m-06-00586-sup1.zip › SeAFFluX user manual.pdf]

SeAFFluX User and Installation Manual

Ryan M. Trevorah
Department of Physics
University of Melbourne

December 7, 2018

1 Introduction

This document details how to use the SeAFFluX program and the necessary installation steps.

2 Installation

- Download the SeAFFluX IDL program and place the '.pro' file in relevant IDL Workspace directory on your computer. The original software is included as Supplementary Information and should always be available from our group's website page:

`http://www.ph.unimelb.edu.au/~chantler/opticshome/softwarepackagedownloads.html`

- Download 'MPFIT' IDL software and place in the same directory as SeAFFluX (or elsewhere as long as you include it in your IDL 'path'):

`http://cow.physics.wisc.edu/~craigm/idl/fitting.html`

- This software uses FFAST tabulated data within the theoretical model. This data is specific to each element, and so the user will have to select whichever data file is appropriate for their model of interest. This data must then be read in to the SeAFFluX software, and interpolated to

create a smooth functional. Note that tabulated data for compounds can also be selected. This data is included in the SeAFFluX package in the 'ffast_files' folder, but can equivalently be downloaded at the following web address:

`https://physics.nist.gov/PhysRefData/FFast/html/form.html`

3 Using SeAFFluX

3.1 Multi-Pixel Detector

This software is currently configured to analyse data from a 36-pixel fluorescence detector. If you want to analyse data from a 100-pixel detector for example, then the size of all relevant arrays must be adjusted accordingly.

3.2 Reading In Data

This software is already configured to read in the data included in the 'Nickel_td_3scans.txt' file. This corresponds to the published fluorescence high-point density i-pr data (Trevorah *et al.*, IUCrJ 2018).
The software also reads in a file called 'iprHPAtrans15mMformatted.txt'. This data is the high-point density transmission XAFS data which was published as Supplementary Information in Chantler *et al.* (JSR 2015). This data is used to assist in maintaining a consistent overall amplitude during the fitting process.

3.3 FFAST Data

FFAST data for all relevant elements/materials considered in your self-absorption model must be read in. It is also likely that a small energy translation will be required to match the peak of the edge in the FFAST data with that of the experimental data considered. It is important to remember to reverse this energy translation after fitting, but before plotting or exporting any of the fitted data.

3.4 Self-Absorption Model

A function must be defined for your theoretical model of self-absorption. This section of the code is well commented, and the underlying theory is discussed in detail in the associated publication. As published, the software first completes an initial fitting pass using the 'linear_seafflux_model' function. This model makes a slight approximation, and so is further corrected by a second pass using an expansion in terms of $[\mu/\rho]^*_{pe}\chi$. The user can select either a quadratic or cubic expansion by toggling the desired function, as described in the comments within the 'self_absorption_chi_model' function.

3.5 Output

A plot is produced at the end of the code as shown in Fig. 1. This shows the correct energy-functional and a small dispersion between spectra.

3.6 Greek Letters in Axis Titles

The example plot shown in SeAFFluX labels the y-axis as 'PLACEHOLDER'. This label is overwritten by the desired $\left[\frac{\mu}{\rho}\right]$ in a subsequent program, the method is outlined at the following link:
`http://www.astrobetter.com/blog/2011/01/04/idl-psfrag/`

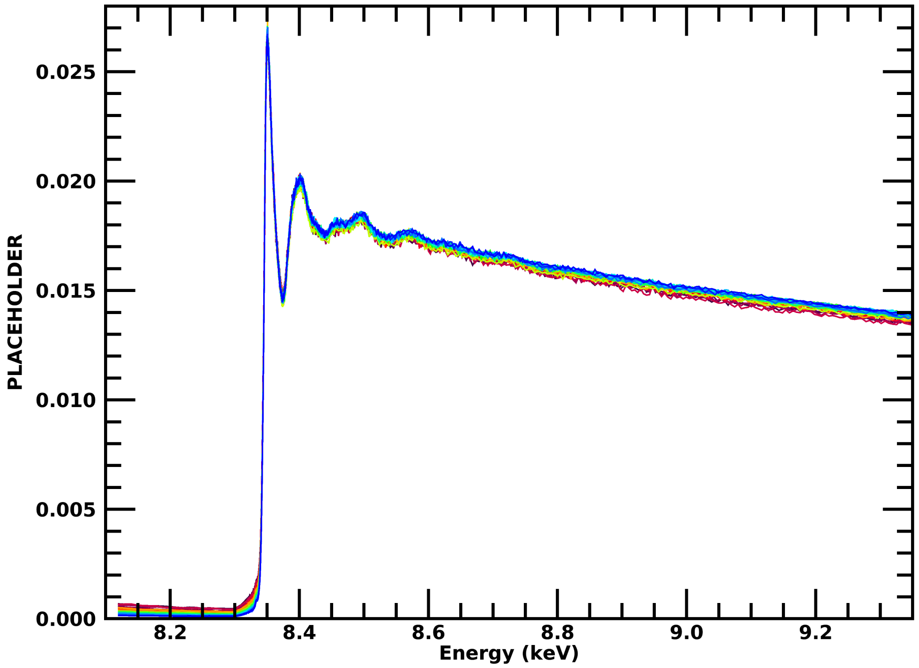

Figure 1: Plot produced at successful completion of SeAFFluX code.
